# Postoperative Adverse Events Inconsistently Improved by the World Health Organization Surgical Safety Checklist: A Systematic Literature Review of 25 Studies

**DOI:** 10.1007/s00268-016-3519-9

**Published:** 2016-04-28

**Authors:** Elzerie de Jager, Chloe McKenna, Lynne Bartlett, Ronny Gunnarsson, Yik-Hong Ho

**Affiliations:** College of Medicine and Dentistry, James Cook University, Townsville, QLD 4814 Australia; College of Public Health, Medical & Veterinary Sciences, The Townsville Hospital, Townsville, QLD 4814 Australia; Cairns Clinical School, College of Medicine and Dentistry, James Cook University, Townsville, QLD Australia; Research and Development Unit, Primary Health Care and Dental Care Narhalsan, Southern Älvsborg County, Region Västra Götaland Sweden; Department of Public Health and Community Medicine, Institute of Medicine, The Sahlgrenska Academy, University of Gothenburg, Gothenburg, Sweden; International College of Surgeons, Chicago, IL USA; Department of Surgery, College of Medicine and Dentistry, James Cook University, Townsville, QLD Australia

## Abstract

**Background:**

The World Health Organization Surgical Safety Checklist (SSC) has been widely implemented in an effort to decrease surgical adverse events.

**Method:**

This systematic literature review examined the effects of the SSC on postoperative outcomes. The review included 25 studies: two randomised controlled trials, 13 prospective and ten retrospective cohort trials. A meta-analysis was not conducted as combining observational studies of heterogeneous quality may be highly biased.

**Results:**

The quality of the studies was largely suboptimal; only four studies had a concurrent control group, many studies were underpowered to examine specific postoperative outcomes and teamwork-training initiatives were often combined with the implementation of the checklist, confounding the results. The effects of the checklist were largely inconsistent. Postoperative complications were examined in 20 studies; complication rates significantly decreased in ten and increased in one. Eighteen studies examined postoperative mortality. Rates significantly decreased in four and increased in one. Postoperative mortality rates were not significantly decreased in any studies in developed nations, whereas they were significantly decreased in 75 % of studies conducted in developing nations.

**Conclusions:**

The checklist may be associated with a decrease in surgical adverse events and this effect seems to be greater in developing nations. With the observed incongruence between specific postoperative outcomes and the overall poor study designs, it is possible that many of the positive changes associated with the use of the checklist were due to temporal changes, confounding factors and publication bias.

## Introduction

One in 25 people undergo a surgical procedure every year [1]. Surgery is intended to save lives but unsafe surgical care can cause substantial harm; complications after inpatient operations occur in 25 % of patients and the reported crude mortality rate after major surgery is 0.5–5 % [2]. At least half of the cases in which surgery leads to harm are considered preventable [3]. Most surgical errors are caused by failures of non-technical skills such as communication, leadership and teamwork [4].

In 2008 the World Health Organization (WHO) developed a surgical safety checklist (SSC), in an attempt to minimise surgical adverse events [2]. The three phase 19-item checklist comprises various perioperative items directly targeted to assure execution of specific safety measures. The mechanism by which the checklist is said to improve surgical outcomes involves both direct and indirect means. Direct factors such as ensuring timely administration of prophylactic antibiotics may result in decreased rates of postoperative infections. Indirectly, the checklist is reported to increase the ‘safety culture’ in operating theatres and thus decrease non-technical surgical errors, resulting in a positive effect on all postoperative adverse events [5–9].

The checklist has been implemented as a standard of care into thousands of operating rooms worldwide as it is relatively easy to implement and unlikely to cause harm [10]. However, there is emerging evidence that for the checklist to be effective it requires a deliberate implementation process, continual monitoring and learning within frontline teams [11]. It is thus necessary to determine the effects of the checklist on postoperative outcomes to validate this continued effort. Furthermore, the checklist may become a routine activity of checking of boxes without actually driving behavioural change thus giving staff a false sense of security [12–14].

Previous literature reviews have all suggested an apparent reduction in postoperative adverse events following the implementation of the checklist; however, all have concluded that higher quality studies are needed [15–21]. Since the last published review, many large-scale studies have been published, including two randomised controlled trials (RCT) [22–26]. Hence there is a need for an updated systematic review of the SSC. This systematic literature review examines the effects of the implementation of the WHO SSC on postoperative complications and mortality.

## Methods

### Protocol and registration

This systematic review is reported using the Preferred Reporting Items for Systematic Reviews and Meta-Analysis (PRISMA) guidelines [27]. The review focuses on studies with primary quantitative data on the effects of the implementation of the WHO SSC on postoperative adverse events. The review was registered in the PROSPERO database, reference number: CRD42015024373.

### Search criteria

A literature search of publications published from 2007 to June 2015 was conducted. Two investigators (EdJ and CM) searched MEDLINE, CINAHL, Scopus, Cochrane and ProQuest databases using the following search strategy; (WHO OR World Health Organisation OR World Health Organization) AND checklist AND (surgery OR surgical OR operative). The date last searched was June 4th 2015. Reference lists of relevant studies were searched by hand to identify additional publications. Authors of select studies were contacted to find additional information. The two investigators screened the titles and abstracts of potential studies, and full text potential studies were reviewed where necessary.

### Eligibility criteria

Included studies incorporated a population of patients undergoing surgical procedures, in which the WHO SSC was implemented, compared to a control group where the checklist was not used or a control group with low compliance to the checklist. The outcomes were quantitative data on postoperative complications or mortality, however defined by the authors. Postoperative pain, urinary tract infections, nausea and vomiting were not considered significant postoperative complications.

Studies were excluded if they were not written in English or did not use the WHO SSC or an adaption of the WHO SSC. Studies were also excluded if the intervention concurrently consisted of a bundle of action such that the sole effect of the safety checklist could not be isolated, for example, where pulse oximetry was introduced alongside the implementation of the checklist.

### Data extraction and analysis

The two investigators used a standardised data sheet to extract data from included studies. Data were extracted for study setting, design and duration, sample size, surgical procedures included and quantitative patient outcomes. Postoperative complication and mortality rates were extracted. Two authors independently performed data extraction and a third review author adjudicated any discrepancies (LB). The included studies were deemed unsuitable for Meta-analysis since they were too heterogeneous and mostly observational studies.

### Quality

Randomised controlled trials were assessed using the Cochrane RevMan Risk of Bias tool [28]. Non-randomised controlled trials were assessed using a modified version of the previously validated Methodological Index for Non-Randomised Studies (MINORS) [29]. The original 12-item index had two items removed by authors, item six and seven. A similar modification has previously been reported [16]. These items relate to an adequate duration of follow-up after the implementation of the checklist. There is currently no consensus about the most appropriate duration of follow-up. There may be an increased emphasis of surgical safety and higher levels of compliance to checklist use early after the intervention, resulting in falsely encouraging outcomes in studies with short follow-up periods. Alternatively, the checklist-induced cultural change may take time to develop and thus studies with a short follow-up period may not show the full effects of the checklists' use. As such, an appropriate length of follow-up could not be defined.

## Results

### Search results

**Fig. 1:**
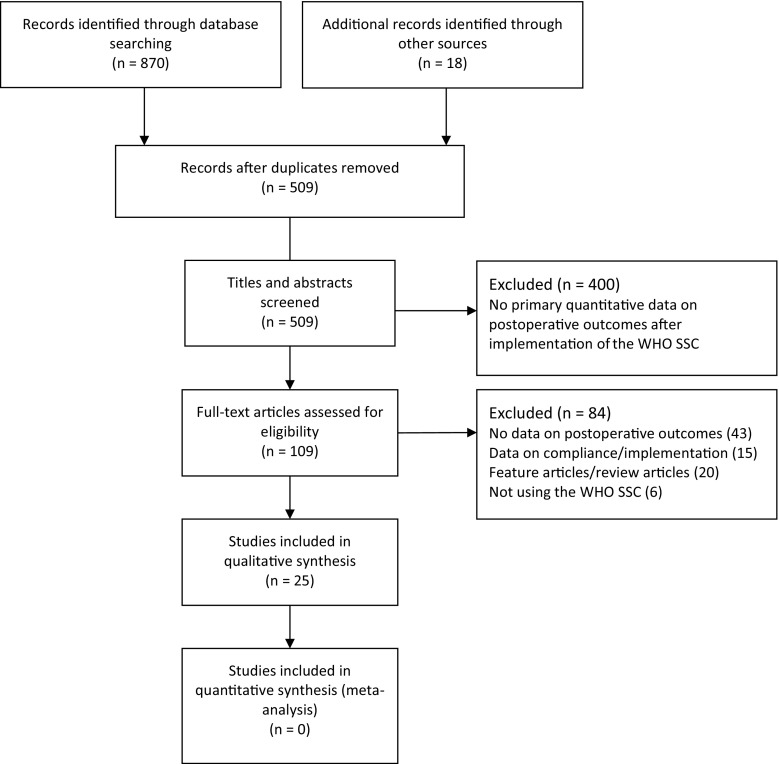
Flow diagram showing identification of studies for inclusion in a systematic review of the effects of the WHO SSC implementation of postoperative adverse events

Database and reference list searches yielded 509 articles, of which full text of 109 articles were examined. Based on the inclusion and exclusion criteria, 25 studies were included (Fig. 1; Table 1) [27].

**Table 1:** Characteristics of included studies (statistically significant results bolded)

Author/year/country (developed nations bolded)	Study Design	Length of review	Sample size	Type of procedures included/excluded	Type of Intervention	Outcome measures	Pre/post, %, *P* value, [% change if significant]
Askarian et al. (2011), Iran [43]	Prospective cohort	Pre: 3 months Post: 3 months	294	Elective general surgery > 16 years		**Total complications**	**Pre: 22.9, Post: 10** ***p*** = **0.03 [-56]**
						SSI	Pre: 10.4, Post: 5.3 *p* = 0.1
						Pneumonia	Pre: 7.6, Post: 3.3 *p* = 0.1
						Acute renal failure	Pre: 4.9, Post: 2.0 *p* = 0.17
Baradaran et al. (2015), Iran [44]	Prospective cohort	NR	200	Elective general surgery > 16 years/end stage & immunocompromised patients		**Any complication**	**Pre: 30, Post 12** ***p*** = **0.002 [-60]**
						**Unplanned readmission to the OT**	**Pre: 9, Post: 2,** ***p*** = **0.03 [-67]**
						SSI	Pre: 13, Post: 7 *p* = 0.157
						Pneumonia	Pre: 8, Post: 3 *p* = 0.121
Biskup et al. (2015), **United states of America** [25]	Retrospective cohort	Pre: 39 months Post: 39 months	4476	Plastic surgery		Total complications	Pre: 5.95, Post: 5.75 *p* = 0.799
						Mortality	Pre: 0.05, Post: 0.04 *p* = 0.549
						Infection	Pre: 1.75, Post: 2.29 *p* = 0.206
						Wound dehiscence	Pre: 1.20, Post: 1.47 *p* = 0.439
						Respiratory failure	Pre: 0.09, Post: 0.04 *p* = 0.613
						Pneumonia	Pre: 0.05, Post: 0 *p* = 0.484
Bliss et al. (2012), **Unites states of America** [33]	Pre: Retrospective historical control Post: Prospective cohort	32 months	2398	Specific high risk electively scheduled procedures, > 18 years/traumatic injuries	Three session team training program	**Any adverse event**	**Pre: 23.6, Post: 8.2** ***p*** = **0.000 [-65]**
						Infectious	Pre: 11.1, Post: 6.8 *p* = 0.514
						Sepsis	Pre: 2.5, Post: 2.7 *p* = 0.355
						Septic shock	Pre: 2.3, Post: 0 *p* = 0.411
						SSI	Pre: 6.2, Post: 5.5 *p* = 0.845
						Pneumonia	Pre: 2.4, Post: 0 *p* = 0.362
						All pulmonary events	Pre: 6.1, Post: 0 *p* = 0.087
						All cardiac events	Pre: 1.9, Post: 0 *p* = 0.124
						**Acute renal failure**	**Pre: 0.4, Post: 0** ***p*** = **0.045 [-100]**
						Bleeding	Pre: 6.1, Post: 2.7 *p* = 0.392
						Surgical wound disruption	Pre: 0.5, Post: 0 *p* = 0.325
						DVT/PE	Pre: 0.7, Post: 0 *p* = 0.074
						Ventilator use > 48 h	Pre: 3, Post: 0 *p* = 0.311
Boaz et al. (2014), Israel [35]	Retrospective review	Pre: 6 months Post: 6 months	760	Adult Orthopaedics		**Mortality**	**Pre: 0.8, Post: 2.7** ***p*** = **0.049 [** **+238]**
						**Total complications**	**Pre 25.9 Post 18.9** ***p*** **=** **0.02 [-27]**
						Length of hospital stay	Pre: 7.3 Post 7.4 *p* = 0.132
						Septic shock	Pre: 0, Post: 0.3 *p* = 0.316
						SIRS	Pre 0.5, Post 0.3 *p* = 0.564
						SSI	Pre: 3.2, Post: 2.1 *p* = 0.368
						**Post operative fever**	**Pre: 10.6, Post: 5.3** ***p*** = **0.008 [-50]**
						**Wound infections at discharge**	**Pre: 0.3, Post: 2.4** ***p*** **=** **0.01 [+700]**
Chaudhary et al. (2015), India [26]	Randomised control trial	13 months	700	Gastroenterology > 16 years		**Mortality**	**Pre: 10, Post: 5.7** ***p*** **=** **0.004 [-43]**
						Complications per patient	Pre: 0.97, Post: 0.8 *p* = 0.06
						**High grade complications per patient**	**Pre: 0.33, Post: 0.23** ***p*** **=** **0.004 [-30]**
						Total complication rate	Pre: 52, Post: 46 *p* = 0.15
						Sepsis	Pre: 30, Post: 26 *p* = 0.31
						**Wound related**	**Pre: 8.5, Post: 4.5** *p* = **0.04 [-47]**
						Respiratory complication	Pre: 3.7, Post: 4 *p* = 1
						Cardiac complication	Pre: 2.5, Post: 3.4 *p* = 0.65
						Renal complication	Pre: 2.2, Post: 0.5 *p* = 0.1
						**Abdominal complication**	**Pre: 28, Post: 19.7** ***p*** = **0.01 [-30]**
						**Bleeding**	**Pre: 2.8, Post: 0.5** ***p*** **=** **0.03 [-82]**
						Length of hospital stay	Pre and Post = 9 days *p* = 0.54
Haughen et al. (2015), **Norway** [23]	Stepped wedge randomised control trial	10 months	5295	Cardiothoracic, neurosurgery, orthopaedic, general & urological	Educational program with standardised lectures and information materials.	Mortality	Pre: 1.6, Post: 1 *p* = 0.151
						**Any complication**	**Pre: 19.9, Post: 11.5** ***p*** = **0.001 [-42]**
						**Unplanned readmission to theatre**	**Pre: 1.7, Post: 0.6** ***p*** **<** **0.001 [-65]**
						**Infectious complications**	**Pre: 6, Post: 3.4** ***p*** **<** **0.001 [-43]**
						Sepsis	Pre: 0.6, Post: 0.3 *p* = 0.075
						SSI	Pre: 2.2, Post: 1.5 *p* = 0.149
						**Pneumonia**	**Pre: 3.7, Post: 1.9** ***p*** **<** **0.001 [-48]**
						**Respiratory complication**	**Pre: 6.4, Post: 3.2** ***p*** **<** **0.001 [-50]**
						**Cardiac complication**	**Pre: 6.4, Post: 4.3** ***p*** **<** **0.004 [-33]**
						**Bleeding**	**Pre: 2.3, Post: 1.2** ***p*** **<** **0.008 [-48]**
						Anaesthetic complication	Pre: 0.3, Post: 0.2 *p* = 0.772
						Embolism	Pre: 0.5, Post: 0.2 *p* = 0.092
Hayes et al. (2009), Multinational [38]	Pre: Retrospective historical control Post: Prospective cohort	Pre: 3 months Post: 3 months	7688		Local study team introduced the checklist using lectures, written materials and direct guidance.	**Total Mortality**	**Pre: 1.5, Post: 0.8** ***p*** = **0.003 [-47]**
						**Any complication**	**Pre: 11, Post: 7** ***p*** **<** **0.001 [-36]**
						**SSI**	**Pre: 6.2, Post: 3.4** ***p*** **<** **0.001 [-45]**
						**Unplanned return to OT**	**Pre: 2.4, Post: 1.8** ***p*** **=** **0.047 [-25]**
						Pneumonia	Pre: 1.1, Post: 1.3 *p* = 0.46
						***India, United states of America, Tanzania, Canada, New Zealand***	No statistically significant changes
						***Jordan***	
						**Mortality**	**Pre: 1, Post: 0** ***p*** **<** **0.05 [-100]**
						**Any complication**	**Pre: 11.6, Post: 7** ***p*** **<** **0.05 [-40]**
						**SSI**	**Pre: 4, Post: 2** ***p*** **<** **0.05 [-50]**
						**Unplanned return to OT**	**Pre: 4.6, Post: 1.8** ***p*** **<** **0.05 [-61]**
						Pneumonia	Pre: 0.8, Post: 1.2 P > 0.05
						***Philippines***	
						**Mortality**	**Pre: 1.4, Post: 0** ***p*** **<** **0.05 [-100]**
						**Any complication**	**Pre: 21.4, Post: 5.5** ***p*** **<** **0.05 [-74]**
						**SSI**	**Pre: 20.5, Post: 3.6** ***p*** **<** **0.05** **[-82]**
						Unplanned return to OT	Pre: 1.4, Post: 1.8 P > 0.05
						Pneumonia	Pre: 0.3, Post: 0 P > 0.05
						***England***	
						Mortality	Pre: 2.1, Post: 1.7 P > 0.05
						**Any complication**	**Pre: 12.4, Post: 8.0** ***p*** **<** **0.05 [-35]**
						**SSI**	**Pre: 9.5, Post: 5.8** ***p*** **<** **0.05 [-38]**
						**Unplanned return to OT**	**Pre: 1.3, Post: 0.2** ***p*** **<** **0.05 [-84]**
						Pneumonia	Pre: 1, Post: 1.7 *p* > 0.05
Jammer et al. (2015), **28 European nations** [51]	Prospective cohort	7 days	45,591 from 426 sites	Non-cardiac surgery > 16 years		No clear relationship between patterns of checklist use and mortality rates in individual countries	
						Crude mortality	Pre: NR, Post: NR *p* = 0.002
						→ Adjusting for confounders	***p*** **<** 0.06
Lepatuma et al. (2013), **Finland** [36]	Pre: Retrospective historical control Post: Prospective cohort	Pre: 6 wks Post: 6 wks	150	Neurosurgery, > 18 years		Total complication	Pre: 58, Post: 46 *p* = 0.16
						**All unplanned readmissions (ICU, OT, hospital 30** **days)**	**Pre: 25.3, Post: 10.4** ***p*** **=** **0.02 [-58]**
						Readmission to operating room	Pre: 19.3, Post: 9 *p* = 0.076
						Infectious	Pre: 13.3, Post: 13.4 *p* = 0.94
						SSI	Pre: 9.6, Post: 4.5 *p* = 0.347
						Pneumonia	Pre: 4.8, Post: 3 *p* = 0.69
						Bleeding	Pre: 14.5, Post: 11.9 *p* = 0.652
						Wound dehiscence	Pre: 3.6, Post: 0 *p* = 0.254
						DVT	Pre: 1.2, Post: 0 *p* = 1
						Mechanical ventilation > 48 h	Pre: 10.8, Post: 7.5 *p* = 0.479
						**Wound combined**	**Pre: 19.3, Post: 7.5** *p* = **0.038 [−61]**
						Duration of hospital stay (Days)	Pre 6.65, Post 6.76 *p* = 0.46
Lubbeke et al. (2013), **France [** 47 **]**	Prospective cohort	Pre: 3 months Post: 3 months immediate 3 months 1 year after 3 months 2 years after	2427	High risk surgical patients > 16 years, ASA 3-5/emergency, gynaecological & obstetric surgery, ambulatory surgery & minor urological surgery		Baseline to post combined	
						Unplanned return to OT	Pre: 7.4, Post: 6 RR = 0.82 CI(0.59-1.14)
						**Unplanned return to OT for SSI**	**Pre: 3, Post: 1.7 RR** **=** **0.56 CI(0.32-1)** **[-43]**
						Unplanned readmission to ICU	Pre: 2.8, Post: 2.6 RR = 0.9 CI(0.52-1.55)
						In hospital mortality	Pre: 4.3, Post: 5.9 RR = 1.44 CI(0.97-2.14)
						Baseline to post period 1	
						Unplanned return to OT	Pre: 7.4, Post: 5.8 *p* = NR
						**Unplanned return to OT for SSI**	**Pre: 3, Post: 1.6** ***p*** = **NR**
						Unplanned readmission to ICU	Pre: 2.8, Post: 3.1 *p* = NR
						**In hospital mortality**	**Pre: 4.3, Post: 7.4** ***p*** = **NR**
						Baseline to post period 2	
						Unplanned return to OT	Pre: 7.4, Post: 6.3 *p* = NR
						**Unplanned return to OT for SSI**	**Pre: 3, Post: 1.6** ***p*** **=** **NR**
						Unplanned readmission to ICU	Pre: 2.8, Post: 2.3 *p* = NR
						In hospital mortality	Pre: 4.3, Post: 4.8 *p* = NR
						Baseline to post period 3	
						Unplanned return to OT	Pre: 7.4, Post: 5.9 *p* = NR
						**Unplanned return to OT for SSI**	**Pre: 3, Post: 1.7** ***p*** = **NR**
						Unplanned readmission to ICU	Pre: 2.8, Post: 2.5 *p* = NR
						In hospital mortality	Pre: 4.3, Post: 5.6 *p* = NR
Mayer et al. (2015), **United Kingdom** [37]	Retrospective review	14 months	6714	General, urological, orthopaedic elective & emergency >16 years	Examined checklist completion vs. not completing the checklist and linked this to postoperative outcomes	Mortality	Pre: 1.4, Post: 0.9 *p* = 0.67
						**Complication**	**Pre: 16.9, Post: 11.2** ***p*** **<** **0.01 [-33]**
Morgan et al. (2015), **United kingdom** [39]	Retrospective review Concurrent control group	Pre: 6 months Post: 6 months	2352	Pre: vascular and general surgery Post: Orthopaedic surgery	One day teamwork-training course, six weekly in service coaching	**Complication rate**	**Pre: 21.5, Post: 26.8** ***p*** = **0.05 [+** **25]** → in concurrent control group during this time period complication rates decreased (27.1 to 25.7)
						**Length of stay**	**Pre: 11.1, Post: 13.2** ***p*** **=** **0.0371 [+** **19]**
						Readmission rate	Pre: 13, Post: 11 *p* = 0.25
Morgan et al. (2015), **United kingdom** [40]	Retrospective review Controlled interrupted time series Concurrent control group	Pre: 6 months Post: 6 months	2221	Elective orthopaedic surgery	Teamwork training, plus training and follow-up support in developing standardised operating procedures	Complication rate	Pre: 14, Post: 18 *p* = 0.33
						Length of stay	Pre: 11, Post: 7.2 *p* = 0.372
						Readmission rate	Pre: 13, Post: 11 *p* = 0.29
Nelson et al. (2014), **United states** [41]	Prospective cohort	3 months	NR	NR		Mortality	No change
						Total complications	No change
Oszvald et al. (2012), **Germany** [52]	Retrospective cohort	Pre: 4 years Post: 18 months	12,390	All neurosurgery cases	Improved compliance to advanced checklist modified to suit local needs and addition of checklist in emergency settings	Number of errors (wrong sided)	Pre: 0.03, Post: 0 *p* = 0.74
Prakash et al. (2014), India [45]	Prospective cohort Concurrent cohort comparison	NR	152	General, obstetrics and gynaecology		**Mortality**	**Pre: 1.38, Post: 0** ***p*** **<** **0.05 [-100]**
						**Total AE**	**Pre: 15.27, Post: 5** ***p*** **<** **0.001 [-67]**
						**SSI**	**Pre: 8.33, Post: 1.25** ***p*** **<** **0.001** **[-85]**
						**Wrong side surgery**	**Pre: 1.38, Post: 0** ***p*** **<** **0.05 [-100]**
						Excessive bleeding	Pre: 1.38, Post: 1.25 P > 0.05
Rodrigo-Rincon et al. (2015), **Spain** [24]	Retrospective cohort	Pre: 12 months Post: 12 months	1602	Adults with a minimum hospital stay of 24 h	22 team training sessions	Mortality	Pre: 1.5, Post: 0.9 *p* = 0.356
						Total complications	Pre: 18.1, Post: 16.2 *p* = 0.35
						Reinterventions	Pre: 5.5, Post: 4.4 *p* = 0.356
						**Total complication rates non- elective**	**Pre: 31.8, Post: 20.4** ***p*** **=** **0.006 [-36]**
						Total complication rates elective	Pre: 12.9, Post 14.7 *p* = 0.42
						**Infections**	**Pre: 13.9, Post: 9.6** ***p*** **=** **0.037 [-31]**
						**Sepsis**	**Pre: 2, Post: 0.5** ***p*** **=** **0.011 [-75]**
						SSI	Pre: 7.1, Post: 6 *p* = 0.419
						Wound disruption	Pre: 4.7, Post: 6.5 *p* = 0.158
						Pneumonia	Pre: 2.8, Post: 1.4 *p* = 0.077
						PE	Pre: 0.1, Post: 0 *p* = 1
						MI	Pre: 0, Post: 0.1 *p* = 0.317
						Renal insufficiency	Pre: 0.05, Post: 0.01 *p* = 0.374
						Bleeding	Pre: 1.5, Post: 1.7 *p* = 0.844
						Thrombophlebitis	Pre: 0.5, Post: 0.4 *p* = 1
						Ventilator use	Pre: 2.2, Post: 1.2 *p* = 0.181
Sewell et al. (2011), **United Kingdom** [6]	Pre: Retrospective historical control Post: Prospective cohort	Pre: 4 months Post: 5 months	965	Orthopaedic procedures	Training video, small and large group education sessions	Mortality	Pre: 1.9, Post: 1.6 P > 0.05
						Total complications	Pre: 8.5, Post: 7.6 P > 0.05
						Unplanned readmission to theatre	Pre: 1, Post: 1 P > 0.05
						SSI	Pre: 4.4, Post: 3.5 P > 0.05
Tillman et al. (2013), **United states** [48]	Retrospective review	Pre: 1 yr Post: 1 yr	6935	Cardiac, colorectal, general, gynaecological, orthopaedic, thoracic & vascular	Multidisciplinary team development, surgical team training, education, monitoring and coaching	Mortality	Pre: 0.9, Post: 1 *p* = 0.79
						SSI	Pre: 3.13, Post: 2.96 *p* = 0.72
						**Colorectal SSI**	**Pre: 24.1, Post: 11.5** ***p*** **<** **0.05 [-52]**
						Orthopaedic SSI	Pre: 1.7, Post: 0.7 *p* = 0.06
						Cardiac SSI	Pre: 7.4, Post: 13.9 *p* = 0.22
						General SSI	Pre: 6.2, Post: 6.1 *p* = 0.92
						Gynaecology SSI	Pre: 2.1, Post: 2.7 *p* = 0.77
						Thoracic SSI	Pre: 2.4, Post: 7 *p* = 0.62
						Vascular SSI	Pre: 2.5, Post: 4.7 *p* = 0.50
Urbach et al. (2014), **Canada** [22]	Retrospective cohort	Pre: 3 months Post: 3 months	215,741 in 101 hospitals	All surgical procedures	Some hospitals used specific intervention or educational programs for the checklist implementation	Mortality	Pre: 0.71, Post: 0.65 *p* = 0.07
						Total complications	Pre: 3.86, Post: 3.82 *p* = 0.53
						**Length of stay (days)**	**Pre: 5.11, Post: 5.07** *p* = **0.003 [-1]**
						**Readmission to theatre**	**Pre: 1.94, Post: 1.78** *p* = **0.001 [-8]**
						Readmission to hospital within 30 days	Pre: 3.11, Post: 3.14 *p* = 0.76
						ED visits in 30 days	Pre: 10.44, Post: 10.55 *p* = 0.37
						Emergency procedure mortality	Pre: 4.51, Post 4.12 *p* = 0.11
						Sepsis	Pre: 0.1, Post: 0.09 *p* = 0.73
						Septic shock	Pre: 0.05 Post 0.05 *p* = 0.83
						Shock	Pre: 0.07 Post 0.09 *p* = 0.26
						SSI	Pre: 0.61, Post: 0.64 *p* = 0.30
						Major wound disruption	Pre: 0.14, Post: 0.13 *p* = 0.61
						Pneumonia	Pre: 0.31, Post: 0.31 *p* = 0.80
						Acute renal failure	Pre: 0.1, Post: 0.13 *p* = 0.08
						Bleeding	Pre: 0.64, Post: 0.63 *p* = 0.76
						**DVT**	**Pre: 0.03, Post: 0.07** ***p*** **<** **0.001 [+** **133]**
						PE	Pre: 0.03, Post: 0.03 *p* = 0.58
						MI	Pre: 0.29 Post: 0.29 *p* = 0.91
						**Ventilator use**	**Pre: 0.08, Post: 0.12** *p* = **0.007 [+** **50]**
Van Klei et al. (2012), **Netherlands** [42]	Retrospective cohort	18 months	25,513	All adult patients that underwent a surgery	Team meeting, compliance monitored monthly	Crude mortality	Pre: 3.13, Post: 2.85 *p* = 0.19
						**Mortality adjusted for baseline differences**	**OR 0.85 CI (0.73-0.98)**
Vats et al. (2010), **United Kingdom** [49]	Pre: Retrospective historical control Post: Prospective cohort	6 months	729	Trauma & orthopaedic, gastrointestinal, gynaecology	Research team meetings with operating theatre staff and local supervision	Mortality	No significant change
						Total complications	No significant change
Weissner et al. (2010), Multinational [50]	Pre: Retrospective historical control Post: Prospective cohort	<12 months	1700	Emergency procedures	Local study team introduced the checklist to the operating room staff through lectures, written materials and direct mentoring	**Mortality**	**Pre: 3.7, Post: 1.4** *p* = **0.0067 [-62]**
						**Total complications**	**Pre: 18.4, Post: 11.7** *p* = **0.0001 [-36]**
						**SSI**	**Pre: 11.2, Post: 6.6** *p* = **0.008 [-41]**
						**Estimated blood loss** **>** **500** **mL**	**Pre: 20.2, Post: 13.2** ***p*** **<** **0.001 [-34]**
Yuan et al. (2012), Liberia [46]	Prospective cohort	Pre: 2 months Post: 2 months	481	>16 years surgical patients	Lectures, written materials, direct guidance, team meetings	***Total***	
						Mortality	Pre: 2.2, Post: 2.8 *p* = 0.334
						**Total complications**	**Pre: 32.9, Post: 19.1** *p* = **0.005 [-42]**
						**SSI**	**Pre: 28.6, Post: 9.9** *p* = **0.001 [-65]**
						***Site 1***	
						Mortality	Pre: 0.9, Post: 4.6 *p* = 0.191
						Total complications	Pre: 16.2, Post: 13.6 *p* = 0.488
						SSI	Pre: 13.1, Post: 9.6 *p* = 0.506
						***Site 2***	
						Mortality	Pre: 3.4, Post: 1.4 *p* = 0.909
						Total complications	Pre: 50, Post: 23.2 *p* = 0.008
						**SSI**	**Pre: 43.4, Post: 10.1** ***p*** **<** **0.001** **[-77]**

Pre = before the intervention, Post = after the intervention, RR = adjusted risk ratio, CI = 95 % confidence interval, SSI = surgical site infection, UTI = urinary tract infection, DVT = deep vein thrombosis, PE = pulmonary embolism, ARF = Acute renal failure, NR = not reported, OT = operating theatre, ED = emergency department, ASA = American Society of Anaesthesiologists score

### Quality assessment

**Fig. 2:**
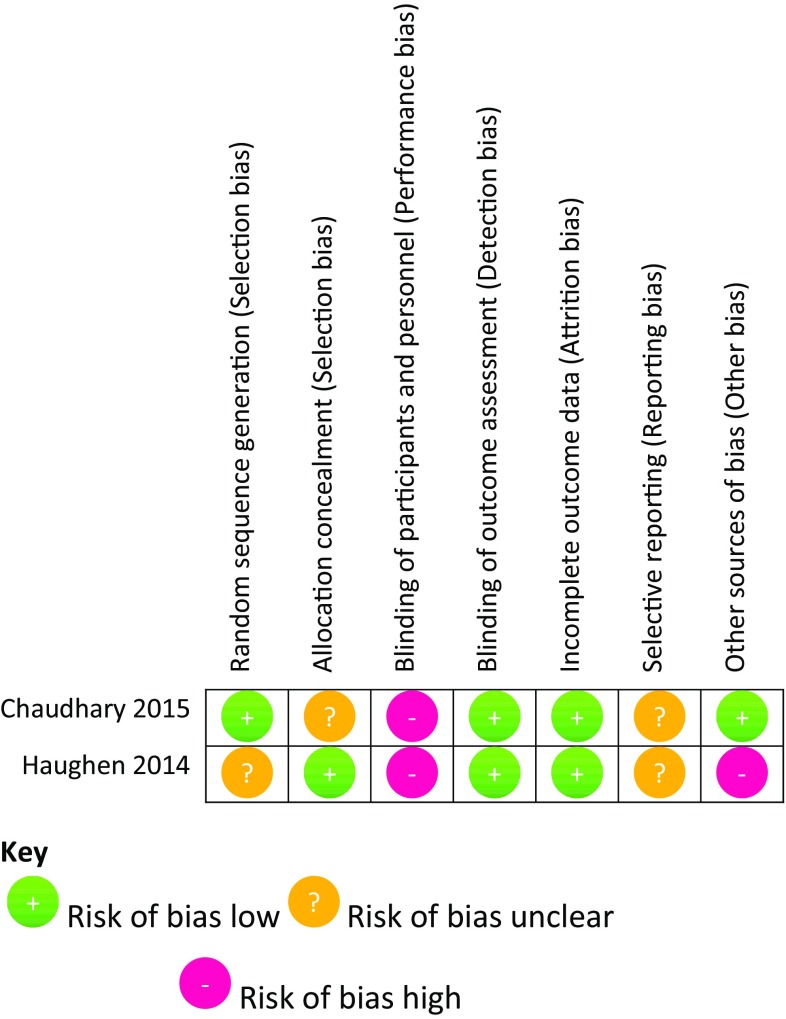
Risk of bias assessment using Cochrane RevMan criteria for randomised controlled studies

**Fig. 3:**
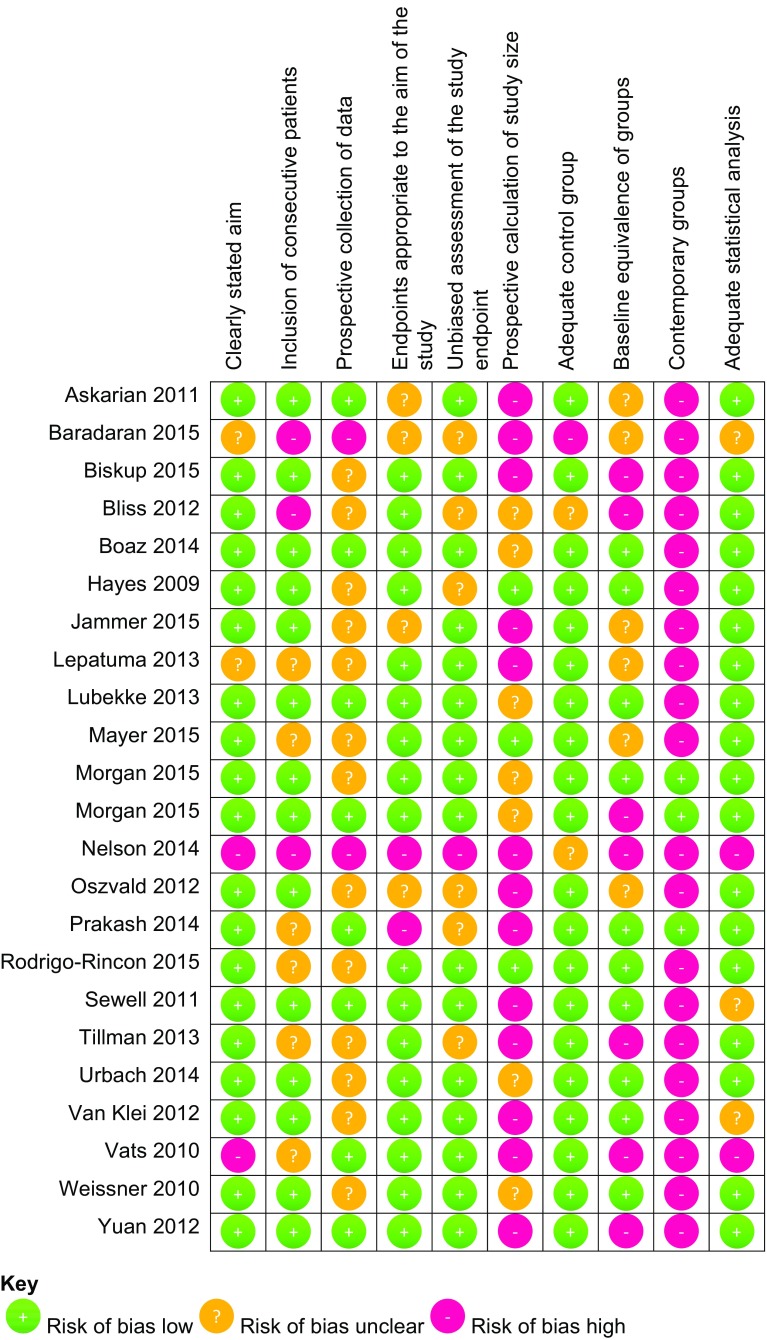
Risk of bias assessment using MINORS criteria for non-randomised studies

Two studies were RCTs, 13 were prospective observational studies and 10 were retrospective cohort studies. The mean Cochrane RevMan score for the two RCTs was nine out of a possible 14. The mean score on the modified MINORS tool was 14 (SD 3.6) out of a possible 20. Each item assessed by these scores may not be equally important. Hence, we refrained from presenting a sum score for individual publications and instead demonstrate the individual components of the scores in a Cochrane risk of bias figure (Figs. 2, 3) [28]. Four studies had a concurrent control group; the remaining studies were largely a pre- and post-implementation group comparison. Several studies did not have adequately matched cohort groups, with differences in the emergency status of the surgery, surgical specialty and patient characteristics.

Many studies did not report doing a sample size calculation. Studies that did do a sample size calculation often calculated these to report significant total pooled complication rates rather than specific postoperative complications. This contributed to many studies being reported underpowered to reach statistical significance for specific postoperative outcomes.

### Risk of bias of included studies

Some generalised potential sources of bias and confounding included that various implementation approaches were used; teamwork-training initiatives themselves may have confounded the post-checklist data [30, 31]. High levels of communication and collaboration are associated with overall lower rates of morbidity [32]. Bliss and colleagues reported a statistically significant decrease in postoperative complications from 23.9 to 15.9 % after three teamwork-training sessions; this was further reduced to 8.2 % after the checklist was adopted [33].

The WHO recommends that local stakeholders alter the checklists. Hence the specific checklists used often vary. This may impact rates of specific postoperative complications and make it difficult to compare studies. The definition of postoperative complications and specific postoperative outcomes also varied between studies making comparison between studies difficult.

Many studies used direct observation to evaluate compliance, potentially leading to a Hawthorne effect where non-technical skills such as communications and leadership increased with the intervention not because of the intervention.

Surgical adverse events rates are influenced by many factors; whilst studies attempted to adjust for known confounders it is likely that there are unknown confounding factors that were not adjusted for. Most of the reviewed studies did not have a concurrent control group and unknown confounding factors likely impacted the interpretation of their results. As the use of the checklist is seen as best practice, it may be unethical to withhold its use in a clinical setting. In addition to this when concurrent control groups are used the contamination effect must be considered, especially for indirect effects of the checklist such as enhanced leadership, teamwork and the resultant improvement in ‘safety culture’.

### Two randomised controlled trials

Chaudhary et al. randomised 700 patients to checklist use or omission in a hospital in India. Patients were blinded to the study whilst the treating teams were not and as such contamination effects may significantly affect the study’s results. Mortality, bleeding, abdominal and wound-related complication rates decreased significantly with the use of the checklist. The total complication rates, number of complications per patient, length of hospital stay, rates of sepsis, respiratory, renal and cardiac complications did not change [26].

A larger stepped wedge cluster randomised control trial with a sample size of 4475 was conducted in two hospitals in Norway. In this study, the checklist intervention was sequentially rolled out across five surgical specialties in a randomised order. As such the cohorts were not adequately controlled; there was a discrepancy in surgical specialty and type of anaesthesia used between cohorts and the intervention group was more likely to undergo emergency surgery. In addition to this, 25.6 % of the procedures allocated to the intervention step were not compliant with the checklist and results of these surgeries were excluded. The reasons for non-compliance were not assessed and this is a likely source of bias. The rates of total complications, unplanned readmission to theatre, infectious complications, pneumonia, haemorrhage, respiratory and cardiac complications significantly decreased, whilst mortality, sepsis, surgical site infections and thromboembolic complications did not significantly change [23].

When results of the two randomised control trials were compared, the only outcome that was significantly decreased in both studies was postoperative bleeding rates.

### Developed vs. developing countries

A sub-analysis was done whereby studies were divided into developing and developed nations as classified by the World Bank classification [34]. Multinational studies that did not differentiate between high- and low-income countries were not included in the sub-analysis. In developed countries, 36 % of studies (5 [23, 33, 35–37] out of 14 studies [6, 22–25, 33, 35–42]) showed a significant decrease in total complication rates compared to 83 % of studies (5 [38, 43–46] out of 6 studies [26, 38, 43–46]) conducted in developing nations. Mortality was not decreased in any of the 13 studies in developed nations [6, 22–25, 35, 37, 38, 41, 42, 47–49], whereas it was decreased in 75 % of studies (3 [26, 38, 45] out of 4 studies [26, 38, 45, 46]) in developing nations. Two studies reported an increase in mortality or complications; both of these studies were in developed nations [35, 39]. Thus in reviewed studies, the effect of the checklist seems to be greater in developing nations.

### Total complications

The total complication rate was reported in 20 studies [6, 22–26, 33, 35–41, 43–46, 49, 50], ten reported significantly decreased rates (range 34–67 %) [23, 33, 35, 37, 38, 43–46, 50] and one reported increased complication rates (25 %) [39].

Mortality rates were reported in 18 studies [6, 22–26, 35, 37, 38, 41, 42, 45–51]; four reported a significant decrease in rates (range 43–100 %) [26, 38, 45, 50], whilst one reported an increase following the implementation of the checklist (238 %) [35].

Length of admission was examined in four studies [22, 26, 39, 40]; one reported a statistically significant but clinically insignificant decrease in length of stay by 0.04 days (*p* = 0.003) [22].

Unplanned return to the operating room was examined in eight studies [6, 22–24, 36, 38, 44, 47]; four found a significant decrease in rates (range 8–67 %) [22, 23, 38, 44].

### Wound related complications

Surgical site infections were examined by 14 studies [6, 22–24, 33, 35, 36, 38, 43–46, 48, 50], four showed a statistically significant decrease (range 41–85 %) [38, 45, 46, 50]. Wound dehiscence was examined by five studies; no significant changes were found [22, 24, 25, 33, 36]. Combined wound complications were examined by two studies; both found a decrease (46 and 61 %) [26, 36].

### Haematological studies

Rates of deep vein thrombosis (DVT) and/or pulmonary embolism (PE) were examined by five studies [22–24, 33, 36]; the only significant change was that one study reported an increase in DVT rates by 133 % [22].

Postoperative bleeding rates were examined by eight studies [22–24, 26, 33, 36, 45, 50]; three found a significant decrease (range 34–82 %) [23, 26, 50].

### Miscellaneous other

Total infection rates were examined in five studies [23–25, 33, 36], rates decreased in two studies [23, 24]. Rates of sepsis were examined in six studies [22–24, 26, 33, 35], rates decreased in one study [24]. Ten studies examined respiratory complications [22–26, 33, 36, 38, 43, 44], one study found a decrease in rates of pneumonia and in total respiratory complication rates [23]. Another study found an increase in ventilation use [22]. Renal complications were examined in five studies [22, 24, 26, 33, 43], one found a decrease in acute renal failure [33], no other results reached significance. Cardiac complications were reported in five studies [22–24, 26, 33], one found a significant decrease in total rates [23]. One study examined total abdominal complications, which showed a reduction in complication rates [26].

### Wrong-sided surgery

Two studies reported rates of wrong-sided procedures [45, 52]. One study found a statistically significant decrease; one patient had a wrong-sided surgery before the implementation, and no patients after the checklist was implemented (1.38 to 0 %, *p* < 0.05) [45].

### Studies with increased rates of adverse outcomes

Two studies showed an increase in postoperative complications and mortality after the implementation of the checklist. In both studies, the comparisons were unadjusted, precluding meaningful conclusions.

Morgan et al. examined the effect of checklist compliance improvement initiatives on surgical outcomes with using a concurrent control group for comparison. In the intervention group, postoperative complications significantly increased, whist in the concurrent control group complications decreased (21.5 to 26.8 and 27.1 to 25.7 %, *p* = 0.05). The study was limited by a small sample size which prevented risk adjustment for differing patient characteristics between the groups. Another limitation was that a direct observational model was used; this is vulnerable to the Hawthorne effect and contamination [39].

Boaz et al. conducted a retrospective review of surgical outcomes before and after implementation of the checklist. It included 760 orthopaedic surgery patients and found an increase in postoperative mortality (0.8 to 2.7 %, *p* = 0.049) following the checklists implementation. The study reported that the composite postoperative complication rates decreased (25.9 to 18.9 %, *p* = 0.02), this was not significant after controlling for confounding variables. The study's conclusion and discussion focussed on a significant decrease in postoperative fever after implementation of the checklist [35].

## Discussion

A surgical safety initiative, which has been implemented into thousands of operating rooms around the world, in an attempt to decrease preventable postoperative complications, should have a strong body of evidence supporting its use. This systematic review found that the effects of the checklist on postoperative outcomes were inconsistent. There may be some benefit to the implementation of the WHO SSC, with this benefit appearing to be greater in developing countries.

There is a lack of significant evidence to explain this phenomenon; that the checklist is more beneficial in developing compared to developed nations. Contributing theories are largely speculative with a lack of significant evidence. Developing countries may have an inherently higher rate of baseline complications and thus have a larger latitude for improvement initiatives to have an effect. Another point to consider is that the checklist partially works by improving non-technical skills such as teamwork, leadership and communication. These factors have a large societal and cultural aspect which may differ between sites. It is also possible that facets of the checklist were already a standard of care in developed countries prior to adoption of the checklist, reducing the effects of the checklist.

Rates of surgical adverse event outcomes are not independent. Postoperative complication rates are associated with postoperative mortality rates [53]. The checklist aims to reduce preventable surgical error and should decrease rates of specific postoperative complications, total surgical complications and postoperative mortality. Outcomes such as the length of stay should also decrease, as these are indirect measures of the postoperative complication rates [54]. The reviewed literature did not show congruency amongst outcomes of surgical adverse event rates. For example, Chaudhary et al. reported that postoperative mortality reduced significantly (by 43 %), whilst there was no significant change in total postoperative complication rates [26]. This phenomenon was observed both within some studies, and when all significant results from the reviewed literature were compared.

An effective safety improvement initiative should have consistent effects on outcomes. The effects of the checklist were inconsistent; this was evident within multicentre studies where the effect of the checklist often varied dramatically between sites. For example, Hayes et al., found significant decreases in postoperative adverse event rates in three of eight sites; the remaining five sites did not have any significant changes in outcomes [38]. The reported benefits of the checklist were from pooled data of all sites. Similarly Urbach et al., examined the effects of the checklist at 101 hospitals, of these six had a significant decrease in adverse event rates, three had a significant increase in adverse event rates and 92 sites had no significant changes in outcomes [22]. Individual sites may not have been sufficiently powered to detect changes, leading to a type two error. Regardless of this factor the effect of the checklist on postoperative outcomes appears to be most variable.

Reviewed studies tended to report substantial improvements in complication rates (range 34–67 %), or show no significant change. Half of surgical complications are reported to be preventable [3]. Hence even if the checklist stopped all preventable errors, postoperative complications would only reduce by 50 %. A change larger than this is likely to have contributing confounding factors or be biased by a poor study design.

Another factor to consider is publication bias. An under-representation of studies showing negative or no effects is well documented; studies with results supporting a hypothesis have a 50 % higher likelihood of publication compared to studies with a negative or neutral outcome [55]. The focus on statistically significant findings was also observed within reviewed studies; with some authors emphasising specific postoperative outcomes that were improved by the checklist, neglecting to comment on the many outcomes that were not altered or increased with the use of the checklist [35].

The checklist may be too generalised as it is intended to be applied to all surgical disciplines. Some specialties have called for their own specific checklists to be created whilst others have proposed a checklist tailored to each specific operation [25, 56–58]. Further studies are needed to determine the effects of specialty-wide surgical safety checklists.

Many of the studies excluded patients below the age of 16 or 18; there is thus a lack of literature reporting the effects of the checklist on a paediatric population. Younger patients may not be able to confirm identity, site or procedure and may lack the ability to give consent. Further studies on the effects of the checklist on a paediatric population are warranted.

A limitation of this review is that reported compliance to the checklist was not scrutinised. Measures of compliance are largely based on specific aspects of care embedded in the checklist. This may be an inappropriate measure of the ‘safety culture’, which the checklist is said to promote. Ticking all the boxes does not mean that the actions the checklist calls for have been completed. Some studies did not report compliance, when it was described there was marked variability in compliance between checklist items [16]. Many studies used data from administrative databases that may report higher rates of compliance than those reported by auditing observers [59, 60]. This heterogeneity makes it difficult to compare compliance rates between studies, and even more so to relate these to adverse event outcome measures in an attempt to draw any meaningful conclusions.

A further limitation is that a meta-analysis was not conducted. Combining observational studies of heterogeneous quality may be highly biased. Included studies had a very diverse patient population and sample size. One study had a larger sample size than all other studies combined, because of this results of a meta-analysis would invariably be skewed to this study’s outcomes.

## Conclusion

The WHO SSC has been widely implemented in an attempt to decrease preventable postoperative complications. This systematic literature review examined the effects of the implementation of the WHO SSC on postoperative adverse events. The review included results of three times as many studies as previously reviewed. The effects of the checklist on postoperative outcomes were inconsistent. With the observed lack of congruency between specific postoperative outcomes and the widespread lack of concurrent control groups, it is possible that many of the positive changes of the checklist were due to temporal changes, rather than the checklist itself. This is likely compounded by publication bias where studies reporting insignificant results are less likely to be published. There may be some benefit to the implementation of the WHO SSC and the benefit appears to be larger in developing countries. Further studies are needed to support the implementation and continued use of the checklist in thousands of operating rooms around the world.
